# Diagnostic Performance of Leukocyte Abnormality Detection in a Large Cohort of Healthy Blood Donors Using Sysmex XN Series Analyzers Integrated with Peripheral Blood Morphology and Flow Cytometry

**DOI:** 10.3390/diagnostics16050661

**Published:** 2026-02-25

**Authors:** Francesca Romano, Valentina Becherucci, Sara Ciullini Mannurita, Edda Russo, Alessandra Mongia, Anna Maria Grazia Gelli, Alessandra Fanelli, Francesca Brugnolo

**Affiliations:** 1 General Laboratory, Azienda Ospedaliero-Universitaria Careggi, 50134 Florence, Italy; romanofr@aou-careggi.toscana.it (F.R.); fanellia@aou-careggi.toscana.it (A.F.); 2 Clinical Pathology Laboratory, Santa Maria Annunziata Hospital, 50134 Florence, Italy; valentina.becherucci@uslcentro.toscana.it; 3 Clinical Pathology Laboratory Unit, S. Giuseppe Hospital, Azienda USL Toscana Centro, 50053 Empoli, Italy; edda.russo@uslcentro.toscana.it (E.R.); annamariagrazia.gelli@uslcentro.toscana.it (A.M.G.G.); 4 Institute for Cancer Research, Prevention and Clinical Network (ISPRO), 50134 Florence, Italy; a.mongia@ispro.toscana.it; 5 Department of Immunohematology, Transfusion Medicine and Laboratory, Meyer Children’s University Hospital, 50134 Florence, Italy; francesca.brugnolo@meyer.it

**Keywords:** WDF channel, WPC channel, blasts, abnormal lymphocytes, atypical lymphocytes

## Abstract

**Background:** The Sysmex XN series (XN-1000 and XN-9100, Sysmex Corporation, Kobe, Japan) represents a latest-generation automated hematology platform integrating fluorescence-based technologies and multi-channel analysis (WDF and WPC) to improve leukocyte characterization. This study aimed to evaluate the performance of the Sysmex XN series in detecting leukocyte abnormalities flagged during routine complete blood count analysis in a large cohort of healthy donors, using morphological assessment and flow cytometry as confirmatory methods. **Methods:** Approximately 8000 healthy blood donors from the AOU Meyer Transfusion Centre were evaluated between 2021 and 2024. All samples underwent CBC analysis using the XN-1000 and XN-9100 analyzers with the WDF channel. Samples showing WBC-related flags were subjected to reflex testing with the WPC channel, followed by digital blood smear review using the DI-60 system (CellaVision, Lund, Sweden) and flow cytometric immunophenotyping. **Results:** WDF flags for “blasts/abnormal lymphocytes” were identified in 23 samples. Two samples were negative on WPC analysis as well as on morphological and flow cytometric evaluation. Among the remaining cases, WPC analysis identified flags for abnormal lymphocytes, atypical lymphocytes, or blasts, which were variably associated with reactive changes, transient immune activation, or clonal lymphoproliferative conditions. In one donor, monoclonal B-cell lymphocytosis was diagnosed by flow cytometry. Overall, reactive morphological features confirmed by flow cytometry were observed in approximately 50% of flagged cases. **Conclusions:** WPC analysis provides relevant additional diagnostic information and demonstrates higher specificity compared with the WDF channel alone; however, it does not fully resolve all instrument-generated flags, confirming the essential role of morphological assessment. Interestingly, the frequent occurrence of inflammatory profiles in recently vaccinated donors suggests that transient immune activation may influence leukocyte flagging. Larger studies are warranted to further investigate this association and to optimize the diagnostic performance of the WPC channel in donor screening.

## 1. Introduction

Over the last decade, substantial technological progress has revolutionized hematological diagnostics through the implementation of high-throughput automated analyzers [1]. These systems have optimized laboratory workflows by delivering rapid, standardized complete blood counts (CBCs) and leukocyte differentials [2]. The progression of these technologies has moved beyond simple cell enumeration to sophisticated multi-parametric analysis such as lineage classification, comprehensive hematological profiling and predictive analytics as well as flagging thanks to alarm signals (“flags”) supporting anomaly detection in a pre-microscopic orientation [3].

Modern automated hematology analyzers, such as the XN series, utilize a multi-modal technological approach to deliver comprehensive diagnostic data. By integrating Electrical Impedance (Coulter Principle), Laser Diffraction, and flow cytometry, these systems transcend basic cell counting to perform complex morphological assessments [4,5]. A distinctive feature of the XN series hematology analyzers is the presence of three specialized measurement channels that utilize Forward Scatter (FSC/FLS), Side Scatter (SSC), and Side Fluorescence Light (SFL) to characterize white blood cell populations (WBCs) [6]. Briefly, the WNR (White Nuclear Red) channel employs FLS and SFL to distinguish Nucleated Red Blood Cells (NRBCs) and Basophils [7], the WDF (White Cell Differential) channel combines SSC (internal complexity) and SFL (nucleic acid/organelle content) to identify atypical lymphocytes and immature granulocytes [8]. Finally the WPC (White Progenitor Cells) channel, available only on the XN-9100, is a specialized reflex module designed to differentiate between malignant blasts and reactive anomalous lymphocytes previously flagged by the WDF channel, by analyzing the relationship between cell size (FSC), granularity (SSC), and metabolic activity (SFL) [9]. The Sysmex XN series utilizes a sophisticated algorithmic approach to manage “suspicious” leukocyte populations detected in the WDF channel generating specific flags [10,11,12] based on the cellular position within the scattergrams, such as ‘Blasts?’, which indicates the probable presence of blasts [13]; ‘Abn Lymph/Blasts?’ indicates cell populations with increased internal cellular complexity frequently associated with lymphoproliferative disorders or severe viral infections [14]; and finally ‘Atyp. Lymph?’, which targets reactive B-lineage cells or significant concentrations (>10%) of large granular lymphocytes (LGLs) [15]. Thanks to this algorithm, the Sysmex XN series helps the clinician provide automated preliminary guidance for the prioritization of manual blood film reviews [16], reducing the turnaround time. Moreover, the WPC classification categories (Reactive, Malignant Suspicion, or Negative) are developed according to the European consensus guidelines for classifying atypical lymphocytes [17]. Furthermore, it has been demonstrated [18] that diagnostic orientation and subsequent investigation can be guided towards clinically similar scenarios merely by analyzing the position of atypical cell populations within the various WPC and WDF channel scattergrams. This capability allows, for example, for the correct differentiation between neoplastic lymphocytosis and reactive lymphocytosis [12]. On the other hand, even if the presence of numerous alarms/flags on WBCs has significantly enhanced the analytical precision and accuracy of the complete blood count, the complexity of this information has necessitated the establishment of stringent rules for count acceptance and microscopic review. In 2005, the International Consensus Group for Hematology Review defined a set of 41 rules for block and microscopic review [19]. The subsequent literature, however, has reported non-concordant performance across different laboratories, largely attributable to variations in analyzer methodologies, procedures for microscopic review, differences in national healthcare organizational models, and laboratory-specific automation workflows [20]. For instance, several surveys have shown that technological and methodological differences among hematology analyzers result in substantial heterogeneity in overall analytical performance, particularly with regard to white blood cell (WBC) and platelet (PLT) counts and morphological flagging [21,22,23,24].

In this context, the debate concerning the sensitivity and specificity of modern automated hematology analyzers remains a crucial topic in laboratory medicine, especially the Clinical Performance and the Role of Flags. An interesting 2002–2003 validation study by the SIPMeL-Italian Society of Clinical Pathology and Laboratory Medicine Working Group (GdS-E) [24], conducted using international NCCLS and ICSH protocols, highlighted the critical role of instrumental “flags”, which led to a notable increase in the negative predictive value (NPV). The conclusion was that differing instrument philosophies and algorithms yield distinct sensitivities and specificities for alarms and instruments, resulting in generally low positive predictive values (PPVs) but consistently high NPVs. This fulfills the primary clinical objective of automation: screening for abnormal samples. Consequently, achieving adequate sensitivity and specificity necessitates the integrated analysis of cell counts, flags, and instrumental scattergrams. This requires a high level of technical expertise and interpretative competence on the part of the laboratory hematologist to achieve the second objective: accurate cellular identification for diagnostic and prognostic purposes [12,25].

In addition, most studies on automated leukocyte abnormality detection are conducted in mixed clinical populations, where the diagnostic focus is the identification of hematologic disease rather than the assessment of physiological normality. Consequently, data derived from large cohorts of healthy blood donors remain limited, despite their relevance for evaluating diagnostic specificity and false-positive rates in routine laboratory practice. Among the few available studies, an evaluation of the Sysmex XN2000 (Sysmex Corporation, Kobe, Japan) analyzer in 262 apparently healthy blood donors reported suspect white blood cell flags in approximately 3% of samples, none of which were confirmed by microscopic examination, indicating a low rate of false-positive flagging in non-pathological conditions [26]. Other investigations involving blood donors have primarily focused on the analytical validation of Sysmex XN series analyzers, demonstrating excellent agreement with previous-generation systems across routine complete blood count parameters, including leukocyte counts [27]. In addition, large population-based studies defining reference intervals and normative ranges using Sysmex XN analyzers provide essential context for interpreting automated leukocyte data under physiological conditions [28,29].

Overall, although evidence on leukocyte abnormality detection in healthy blood donor cohorts is limited, existing studies underscore the importance of this population as a diagnostic reference. This gap supports the need for comprehensive diagnostic evaluations integrating automated analyzers with peripheral blood morphology and flow cytometry.

This study aimed to evaluate and directly compare the performance of the Sysmex XN-1000 and XN-9100 analyzers in terms of detecting leukocyte abnormalities flagged during routine complete blood count analysis. The novelty of this work lies in the head-to-head assessment of automated WBC flagging in a large cohort of healthy blood donors, using a standardized confirmatory workflow that integrates reflex testing with the WPC channel, digital blood smear review, and flow cytometry. This design provides real-world evidence on the reliability and clinical utility of leukocyte flags in a transfusion medicine setting.

## 2. Materials and Methods

### 2.1. Sample Collection

The study was conducted on samples analyzed during routine laboratory workflow between 2021 and 2024, comprising a total of 8000 complete blood counts. Samples were obtained from the blood donation outpatient clinic and represented a theoretically healthy adult population aged 18 to 65 years.

### 2.2. Whole Blood Count

**Figure 1:**
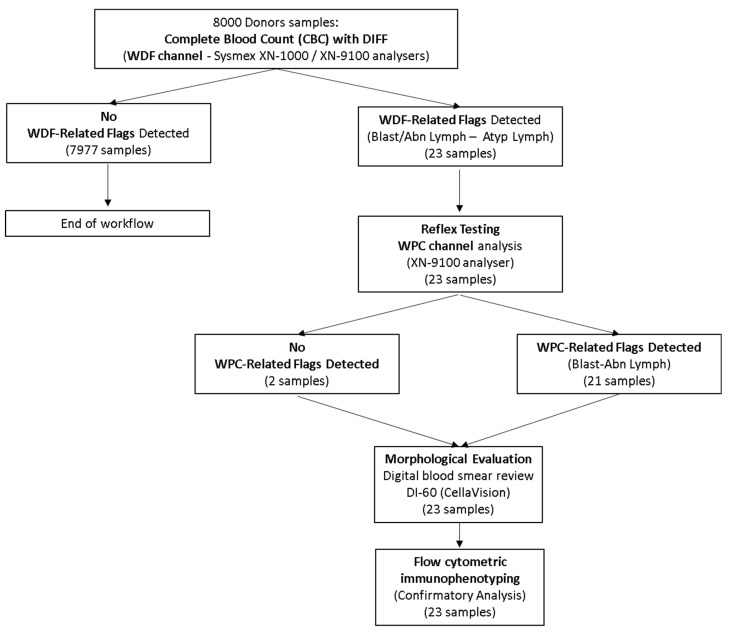
Operational workflow of the study. A total of 8000 healthy donors underwent complete blood count analysis using Sysmex XN automated hematology analyzers, with leukocyte differential counts performed through the WDF channel. Instrument-generated flags on the WDF channel were observed in 23 cases. These samples were subjected to further evaluation using the WPC channel. In 2 cases, the repeat analysis yielded negative results, whereas in the remaining 21 cases the WPC-related flags were positive. All 23 samples underwent peripheral blood smear examination and flow cytometric analysis.

Samples were collected in K3-EDTA plastic Vacutainer™ tubes (Becton, Dickinson and Company, BD, Franklin Lakes, NJ, USA) in accordance with current pre-analytical procedures and protocols, and were processed within 6 h of collection using the Sysmex XN-1000 and XN-9100 hematology analyzers (Sysmex Corporation, Kobe, Japan). Q-flag and alarm message settings were maintained at the default values recommended by the manufacturer. All samples underwent standard complete blood count and leukocyte differential analysis (CBC-DIFF) in ‘WHOLE BLOOD’ operating mode at Meyer Children’s Hospital. All samples exhibiting WBC-related flags (*n* = 23) were subjected to reflex analysis using the WPC channel at AOU Careggi, followed by microscopic blood smear review using the DI-60 digital cell analyzer (CellaVision) (Figure 1).

### 2.3. Flow Cytometry Analysis

Comprehensive flow cytometry immunophenotyping was performed at AOU Meyer on all 23 samples exhibiting WBC-related flags. Data acquisition was carried out using a BD FACSCanto™ II flow cytometer equipped with BD FACSDiva™ 2.0 software. For each sample, three tubes containing 100 μL of whole blood were prepared and incubated for 15 min in the dark at room temperature with specific antibody cocktails (BD Bioscience, San Jose, CA, USA). Antibody panels were designed in accordance with European guidelines for the diagnosis of hematological malignancies [30] and the European Society for Clinical Cell Analysis guidelines [31], as follows:

- Tube 1 (B-cell Compartment): CD27 FITC, CD5 PE, CD45 PerCP-Cy5, CD10 Pe-Cy7, CD19 APC, CD20 APC-Cy7, focusing on B-cell pathologies (e.g., Follicular Lymphoma and Chronic Lymphocytic Leukemia).
- Tube 2 (T-cell Compartment): CD4 FITC, CD8 PE, CD2 PerCP-Cy5, HLA-DR Pe-Cy7, CD3 APC, CD45 APC-Cy7, focusing on T-cell analysis and activation status.
- Tube 3 (Monocyte/NK Compartment): CD16 FITC, CD56 PE, CD45 PerCP-Cy5, HLA-DR Pe-Cy7, CD3 APC, CD14 APC-Cy7, focusing on NK cell and monocyte activation and subtyping (M1, M2, and M3 populations).

After incubation, samples were treated with diluted BD FACS™ Lysing Solution (BD Biosciences) for 10 min in the dark at room temperature to lyse erythrocytes. The samples were then centrifuged at 1500× *g* for 10 min. The supernatant was discarded, and the remaining cell pellet was resuspended in 1 mL of phosphate-buffered saline (PBS; 10×, Euroclone S.p.A.) prior to data acquisition on the flow cytometer.

### 2.4. Statistical Analysis

True positives (TPs) were defined as samples showing concordant positivity on both morphological examination and flow cytometric analysis. False positives (FPs) were samples flagged by the analyzer but not confirmed by either morphology or flow cytometry. True negatives (TNs) were unflagged samples confirmed as negative, whereas false negatives (FNs) were samples not flagged by the analyzer but demonstrating positivity on morphological or flow cytometric evaluation. In the overall cohort of 8000 donors, for analysis of the WDF channel on the XN-1000 platform, positive flags were defined as instrument-generated alerts for blasts/abnormal lymphocytes or atypical lymphocytes, while all other samples without flags were classified as negative. For the WPC channel on the XN-9100 platform, positive flags included abnormal lymphocytes, blasts, or atypical lymphocytes, whereas negative flags comprised the remaining samples. Diagnostic performance metrics were calculated using standard formulas for diagnostic test evaluation. Statistical analyses were performed using Microsoft Excel.

## 3. Results

**Figure 2:**
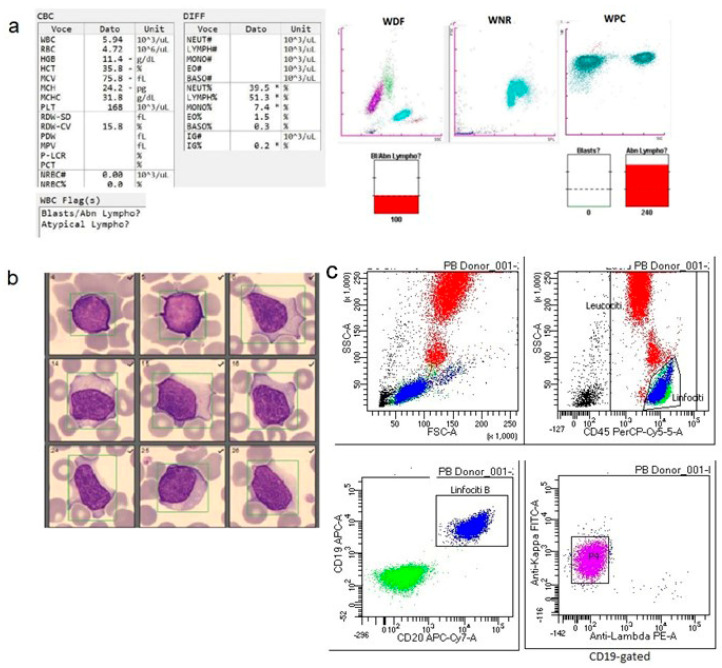
Monoclonal B-cell Lymphocytosis (MBL). (**a**) Complete blood count (CBC) and scattergram with corresponding values and alarm flags, performed on a Sysmex XN-9100. * = out of range value (**b**) peripheral blood smear showing the presence of atypical lymphocytes (May–Grünwald Giemsa stain, ×100); (**c**) flow cytometry dot plots confirming and further characterizing non-CLL-like monoclonal B-cell lymphocytosis, showing an inverted leukocyte differential, expansion of the B-lymphocyte population (60% of total lymphocytes), and weak-intensity clonal κ surface light-chain expression in 98.9% of B cells.

**Figure 3:**
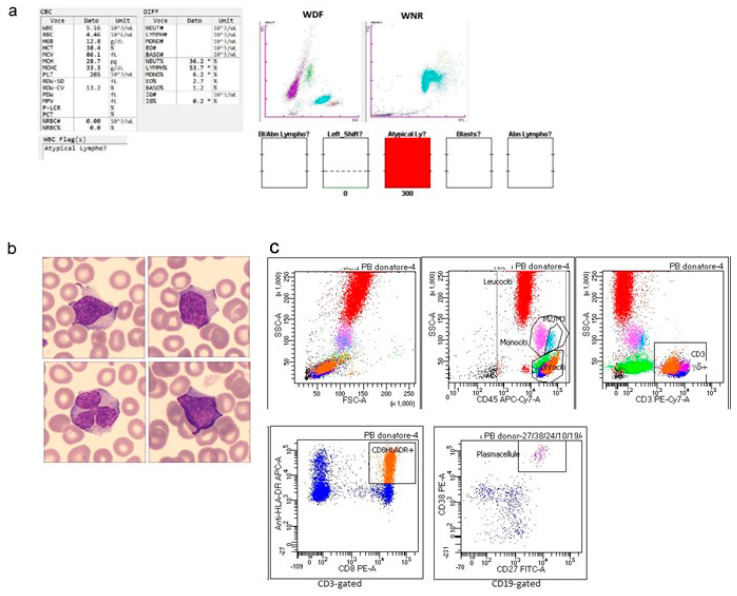
Inflammatory state with increased plasmacytes and γδ T lymphocytes. (**a**) Complete blood count (CBC) and scattergram with corresponding values and alarm flags, performed on a Sysmex XN-9100; * = out of range value (**b**) peripheral blood smear showing the presence of activated lymphocytes (May–Grünwald Giemsa stain, ×100); (**c**) flow cytometry dot plots confirming circulating plasmablast expansion (1.5% of total lymphocytes), increased γδ T lymphocytes, the presence of HLA-DR-positive CD8 T cells, and an elevated proportion of M2–M3 monocytes (the P5 and P6 gates, respectively).

Approximately 8000 healthy blood donors were evaluated between 2021 and 2024. Only 23 samples (0.0029%) triggered “blasts/abnormal lymphocytes” flags on the WDF channel of the Sysmex XN1000 and XN9100 analyzers (Table 1). In the 23 analyzed samples, the mean total white blood cell count was 8.9 × 10^9^/L, with the following corresponding mean values: neutrophils 3.7 × 10^9^/L, lymphocytes 3.8 × 10^9^/L, and monocytes 0.6 × 10^9^/L (Table 2). Two samples were completely negative on both the WPC channel, as well as on morphological and flow cytometric analyses. In the remaining 21 donors, the WPC channel displayed the following flags: “abnormal lymphocytes” in 13 samples. Among these, in one donor the microscopic review confirmed the presence of atypical cells and Gumprecht shadows, and flow cytometry established a diagnosis of monoclonal B-cell lymphocytosis (Figure 2). Among the remaining samples, five were negative on both morphological examination and flow cytometry. One sample exhibited large granular lymphocytes on peripheral blood smear but was negative by flow cytometric analysis. One sample was morphologically negative but showed circulating plasmablasts by flow cytometry. Two samples displayed activated lymphocytes on peripheral blood smear; in one of these, flow cytometry identified HLA-DR-positive and γδ-positive T lymphocytes together with an increased proportion of M2–M3 monocytes (Figure 3). In the other case, flow cytometric analysis also confirmed the presence of activated lymphocytes. According to the donor’s medical history, the individual had experienced an episode of acute, undiagnosed infection one week prior to donation (patient 8, Table 1).

Moreover, in another case, an inverted leukocyte differential count and large granular lymphocytes were observed on morphology, and flow cytometry showed an increase in γδ+ T cells, CD8-positive T cells, and NK cells. In another sample, large granular lymphocytes were observed morphologically, whereas flow cytometry revealed an inversion of the CD4/CD8 ratio and an increase in the M2–M3 monocyte subpopulation. In an additional donor, morphology showed an inverted leukocyte differential count, whereas flow cytometry revealed an expansion of NK cells.

Two samples displayed a ‘blasts’ flag on the WPC channel. One case was negative on both morphological and flow cytometric analyses, whereas the other showed activated lymphocytes on morphological examination and an increased proportion of NK cells and M3 monocytes by flow cytometry.

Three samples showed the “atypical lymphocytes” flag on the WPC channel. Among these, one sample had also triggered the “blasts/abnormal lymphocytes” flag on the WDF channel on both analytical platforms (XN1000 and XN9100), whereas the other two showed only the “atypical lymphocytes” flag on WDF. In these three cases, two showed lymphoplasmacytoid lymphocytes on morphological examination, and flow cytometry confirmed the presence of circulating plasmablasts in both, and an increase in γδ T cells was observed in only one of them. In the third case with a WPC “atypical lymphocytes” flag, activated lymphocytes were observed on peripheral smear, and flow cytometry identified an increase in γδ+, CD8-positive T lymphocytes with an inverted CD4/CD8 ratio. According to the patient’s post-donation information, 2 weeks before donation, he experienced an episode of acute infection, then diagnosed as EBV infection (patient 15, Table 1).

Among the remaining samples, one donor showed a “blasts/abnormal lymphocytes” flag on the XN1000 WDF channel, whereas both WDF and WPC channels on the XN9100 were negative. However, morphological analysis was negative, while flow cytometry demonstrated an increase in γδ T lymphocytes and activated monocytes. In another case, the WDF channels on both platforms showed the “blasts/abnormal lymphocytes” flag, whereas the XN9100 WPC channel was negative, but morphological analyses showed activated lymphocytes, confirmed by flow cytometry through the identification of γδ T cells. In the last case, the WDF channel on both platforms showed the “blasts/abnormal lymphocytes” flag, while the WPC channel was negative, but morphological evaluation demonstrated lymphoplasmacytoid lymphocytes, confirmed by flow cytometry as plasmablasts.

**Figure 4:**
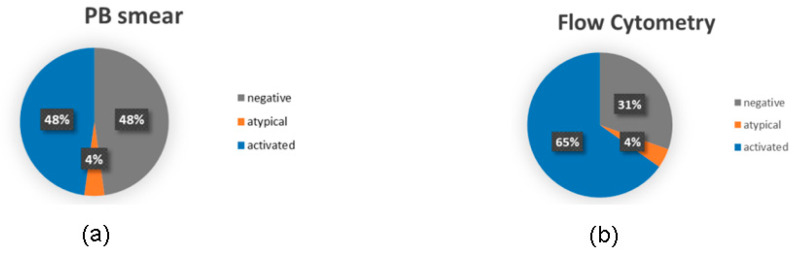
Description as a percentage of flag-positive samples through morphological evaluation (**a**) and flow cytometric analysis (**b**).

Overall, 50% of flagged cases exhibited reactive morphological features on peripheral blood smear that were corroborated by flow cytometry. The case diagnosed as monoclonal B-cell lymphocytosis (MBL) should be considered atypical, whereas all other non-normal cases were classified as reactive (Figure 4a,b).

In the overall cohort of 8000 donors, 23 samples were positive on the WDF channel of the XN-1000 platform, of which 12 showed concordant positivity on both morphological examination and flow cytometric analysis. Eleven samples displayed instrument-generated positive flags without confirmation by either morphology or flow cytometry.

We performed the same analysis on samples testing positive on the WPC channel of the XN9100 platform. In this case, we observed 18 samples with positive flags, while 5 samples were negative.

## 4. Discussion

Over the last decade, fully automated hematology analyzers have evolved significantly, enabling rapid acquisition of a complete blood count with differential. The Sysmex XN series, utilizing fluorescence-based technology and dual-channel analysis (WDF and WPC), provides enhanced leukocyte characterization compared to conventional single-channel systems [32,33].

Our data indicate that the WPC channel provides substantial diagnostic benefit by reducing false-positive results, halving the number of misleading ‘blasts/abnormal lymphocytes’ flags compared with use of the WDF channel alone. In line with previously published studies, integration of this approach with automated slide review using the DI-60 system markedly streamlines laboratory workflow, achieving an approximately 40% reduction in manual slide review [33,34,35,36].

Nevertheless, instrument-generated flags do not provide definitive diagnostic interpretation, and morphological assessment remains essential for the resolution of ambiguous cases.

Only through the integrated interpretation of scattergram analysis (WDF/WPC), morphological assessment, and flow cytometry can reactive, preneoplastic, and truly pathological cell populations be reliably distinguished [34,37]. Moreover, the heterogeneity observed among flagged cases indicates that some donors may harbor clinically relevant abnormalities despite normal leukocyte counts. Notably, reflex testing enabled the identification of cases consistent with monoclonal B-cell lymphocytosis (MBL) as well as reactive lymphocyte activation that might otherwise have gone unrecognized. While the detection of MBL in this context is clinically noteworthy, its implications should be interpreted cautiously, particularly in an asymptomatic healthy donor population. The clinical significance of incidentally identified low-count MBL remains variable, and these findings primarily underscore the potential of extended diagnostic workflows to reveal subclinical hematologic alterations rather than supporting routine screening for such conditions.

Interestingly, all 23 donors who triggered instrument-generated flags had received SARS-CoV-2 vaccination within 20 days prior to donation. In addition, two of these donors reported a recent infectious episode: one with a documented recent Epstein–Barr virus (EBV) infection and the other with an acute infection of unidentified etiology.

The observation that several cases exhibiting an “inflammatory profile” occurred in donors sampled shortly after vaccination provides a potentially relevant clinical context. However, this temporal association should be interpreted with caution and does not establish a causal relationship between vaccination and the observed leukocyte pattern alterations. It is plausible that transient immunological activation—whether related to recent vaccination, intercurrent infection, or other stimuli—may influence leukocyte distributions and contribute to instrument-generated flags. Nevertheless, definitive conclusions cannot be drawn from the present data. Prospective studies with appropriate controls and standardized sampling times are needed to clarify the clinical relevance of these findings and to prevent overinterpretation of reactive laboratory profiles [34].

These results indicate that both platforms exhibit negative predictive value, suggesting that instrument-generated flags are highly reliable for ruling out abnormal or atypical lymphocyte populations. The slightly lower positive predictive value reflects the occurrence of false-positive flags, underscoring the continued need for morphological review and, when appropriate, flow cytometric confirmation in flagged cases.

The difference in positive predictive value between the XN-1000 and XN-9100 platforms may reflect platform-specific thresholds for flag generation as well as differences in channel configuration (WDF vs. WPC). Importantly, the consistently high negative predictive value observed across both platforms supports the use of these automated systems as effective screening tools in large donor populations, minimizing the risk of missing clinically relevant abnormalities while reducing unnecessary manual reviews.

However, beyond the limitations already discussed, this study presents some additional constraints. It was conducted in a single-center setting, which may limit the generalizability of the findings, as donor selection criteria and laboratory practices can vary across institutions. Additionally, the demographic characteristics of our donor population may not fully reflect broader or more diverse populations. Finally, because the cohort consisted of asymptomatic, pre-screened healthy donors, the prevalence and clinical relevance of incidental hematologic findings should be interpreted with caution. Multicenter studies would be valuable to confirm the wider applicability of these results.

Overall, our findings underscore the complementary roles of instrument-generated flags, morphological assessment, and flow cytometry in the accurate identification of reactive, preneoplastic, and pathological lymphocyte populations, supporting the adoption of an integrated diagnostic approach in routine hematology practice.

## 5. Conclusions

In summary, this study demonstrates the robust diagnostic performance of the Sysmex XN-1000 and XN-9100 platforms for the detection of leukocyte abnormalities in a large cohort of blood donors. Our findings highlight the added value of the WPC module, which significantly reduces false-positive flags generated by the WDF channel. Although WPC analysis provides critical additional information on abnormal cell populations, it does not fully resolve all instrument-generated alerts, underscoring the indispensable role of morphological assessment in differentiating reactive from pathological findings. The integrated diagnostic workflow combining WDF and WPC scattergram analysis with automated morphological review demonstrated high diagnostic accuracy while substantially reducing the burden of manual slide review. Importantly, even within a theoretically healthy donor population, instrument-generated flags may reflect clinically relevant conditions, such as monoclonal B-cell lymphocytosis, or transient immune activation related to recent infections or vaccination, emphasizing the need for careful clinical and laboratory interpretation. Morphological evaluation and scattergram interpretation therefore remain essential complements to automated flagging systems, guiding appropriate reflex flow cytometric investigations and ensuring accurate classification of leukocyte abnormalities. The observation that all donors with positive WDF flags had received SARS-CoV-2 vaccination within 20–30 days prior to donation suggests that recent immunization may influence leukocyte patterns and contribute to reactive profiles; however, causality cannot be inferred from the present data, and further investigation is warranted. Future studies should include larger, longitudinal cohorts and refined platform-specific algorithms to further improve the diagnostic performance of automated leukocyte flagging. A standardized multimodal approach integrating automated analysis, morphology, and flow cytometry may enhance early detection of subtle abnormalities, optimize laboratory resources, and improve donor safety.

## Figures and Tables

**Table 1:** Characteristics of Donors Exhibiting WBC-Related Flags Included in the Study.

Sample	WDF XN-1000	WDF XN-9100	WPC XN-9100	PB Smear	Flow Cytometry
1	blasts/abn lympho	blasts/abn lympho	Negative	Normal	Negative
2	blasts/abn lympho	Negative	Negative	Normal	Increased γδ T lymphocytes, activated monocytes
3	blasts/abn lympho	blasts/abn lympho	abn lympho	Normal	Negative
4	blasts/abn lympho	blasts/abn lympho	abn lympho	Normal	Negative
5	blasts/abn lympho	blasts/abn lympho	blasts	Normal	Negative
6	blasts/abn lympho	blasts/abn lympho	abn lympho	Large granular lymphocytes, inverted leucocyte differential count	Increased γδ T lymphocytes, CD8 T lymphocytes and NK cells
7	blasts/abn lympho	blasts/abn lympho	abn lympho	Normal	Negative
8	blasts/abn lympho	blasts/abn lympho	abn lympho	Activated lymphocytes	Increased CD8+ HLA-DR+, γδ T cells and increased Monocyte M2-M3 subpopulations
9	blasts/abn lympho	blasts/abn lympho	abn lympho	Normal	Negative
10	blasts/abn lympho	blasts/abn lympho	blasts	Activated lymphocytes	Increased Monocytes (M3), and NK cells
11	blasts/abn lympho	Negative	negative	Normal	Negative
12	blasts/abn lympho	blasts/abn lympho	abn lympho	Large granular lymphocytes	Negative
13	blasts/abn lympho	blasts/abn lympho	abn lympho	Activated lymphocytes	Activated lymphocytes
14	blasts/abn lympho	blasts/abn lympho	abn lympho	Athypical lymphocytes and smudge cells	Monoclonal B LLC-like MBL
15	atypical lympho	atypical lympho	atypical lympho	Activated lymphocytes	Increased CD8 HLA-DR+ and γδ T cells, inverted CD4/CD8 ratio
16	blasts/abn lympho	blasts/abn lympho	abn lympho	Normal	Increased circulating plasmablasts
17	blasts/abn lympho	blasts/abn lympho	abn lympho	Normal	Negative
18	blasts/abn lympho	blasts/abn lympho	Negative	Activated lymphocytes	Increased γδ T lymphocytes
19	blasts/abn lympho	blasts/abn lympho	atypical lympho	Lymphoplasmacytoid lymphocytes	Increased circulating plasmablasts and γδ T lymphocytes
20	blasts/abn lympho	blasts/abn lympho	abn lympho	Large granular lymphocytes	Increased Monocyte M2-M3 subpopulations, inverted CD4/CD8 ratio
21	atypical lympho	atypical lympho	atypical lympho	Lymphoplasmacytoid lymphocytes	Increased circulating plasmablasts
22	blasts/abn lympho	blasts/abn lympho	abn lympho	Inverted leucocyte differential count	Increased NK cells
23	blasts/abn lympho	blasts/abn lympho	Negative	Lyphoplasmacytoid lymphocytes	Increased circulating plasmablasts

**Table 2:** CBC and differential in flag-positive donors enrolled in the study.

	WBC (10^9^/L)	NEUT (%)	LYMPH (%)	MONO (%)	NEUT (10^9^/L)	LYMPH (10^9^/L)	MONO (10^9^/L)
Donors mean	8.90	43.16	46.24	7.02	3.70	3.84	0.59
Donors SD	2.10	10.31	10.33	1.69	1.50	1.14	0.19
Reference values	4.00–10.00	37–75	12–50	3–12	1.0–7.50	0.50–5.00	0.03–1.20

## Data Availability

The datasets analyzed during the current study are available on reasonable request.

## Abbreviations

The following abbreviations are used in this manuscript:

WDF	White differential fraction
WPC	White progenitor cell
CBC	Cell blood count
MBL	Monoclonal B-cell lymphocytosis
PPV	Positive predicted value
NPV	Negative predicted value
